# The Relationship Between School Bullying and Subjective Well-Being: The Mediating Effect of School Belonging

**DOI:** 10.3389/fpsyg.2021.725542

**Published:** 2021-09-20

**Authors:** Zhuzhu Xu, Chenchen Fang

**Affiliations:** Graduate School of Education, Peking University, Beijing, China

**Keywords:** mediating effect, PISA2018, school belonging, school bullying, subjective well-being

## Abstract

In order to deepen people's understanding of how school bullying influences subjective well-being of students involved, this research system explores the relationship between school bullying, school belonging, and subjective well-being, and the data of students in mainland China (represented by Beijing, Shanghai, Jiangsu, and Zhejiang) participating in the PISA 2018 test were used for analysis. The results show that school bullying has a significant negative correlation between students' school belonging and subjective well-being. Besides, school belonging plays a part role in mediating the negative correlation between school bullying and subjective well-being of middle school.

## Introduction

In the past decade, the research on school bullying has been on the rise, and European and American scholars have dominated the research, while China and other Asian regions have slightly insufficient attention to this area (Li et al., 2017), especially in the context of Chinese, research on how school bullying influences students' subjective well-being is even rarer (Guo, 2019). This situation may be stem from many reasons. On the one hand, the school education in mainland China generally values students' academic performance, learning ability, and other cognitive performance, while less attention is paid to campus safety environment and students' emotional attitudes. On the other hand, the school is the main place where the country exerts educational influence, and the positive emotional experience in students' daily life, such as school belonging, actually constitutes the direct source of their subjective well-being, thus neglecting the indirect role of school belonging in buffering the impact of school bullying on subjective well-being. Moreover, compared with foreign studies, Chinese school education emphasizes the shaping of individual behaviors from the collective, and this collectivism requires students to identify with the class or school and other collectives (Cortina et al., 2017). Therefore, when students experience bullying on campus, those students who are recognized by their peers and love the group will often receive timely psychological support, and the other part of students who no longer trust and recognize classes and schools will become more isolated and loss, and thus their satisfaction with school life is declining (Adams and Hannum, 2016). So in the context of Chinese culture, school belonging may involve in alleviating the negative impact of school bullying on subjective well-being.

Furthermore, although the international community has conducted a large number of investigations and empirical studies on school bullying in major countries or economies in the world, more attention is paid to the impact of school bullying, and subjective well-being on academic performance (Nakamoto and Schwartz, 2010) or simply analyze the direct impact of school bullying on students' subjective well-being (Tiliouine and Habib, 2015). And so far, few studies have considered how both school bullying and school belonging influence the subjective well-being of students. In addition, from the reality of China, although the frequency of school bullying in mainland China has not been as serious as in other countries or regions in recent years, the international survey results show that Chinese student are not happy at school, and their sense of school belonging and satisfaction are lower (OECD, 2017, 2019). As a result, it is necessary for the academic community to clarify the potential mechanism between school bullying behavior and students' subjective well-being in mainland China. On the one hand, these studies can help to enhance the subjective well-being of Chinese students at school; on the other hand, it can also better provide useful information for the Chinese education management department to formulate effective anti-bullying policies.

## Literature Research

In recent years, school bullying has turned into a major threat in campus safety around the world. It not only has a serious impact on students' daily life, but also increasingly erodes the students' original well-being. The literature on the relationship between school bullying and subjective well-being shows that school bullying seems to inhibit students' subjective well-being (Woods and Wolke, 2004; Chrysanthou and Vasilakis, 2019). Some research reports indicate that the subjective well-being of adolescents who have suffered various injuries such as bullying is significantly lower than average, and the direct impact of violence such as school bullying on subjective well-being far exceeds other indicators (Pople et al., 2015). In addition, some studies further found that long-term bullying experience will gradually increase negative feelings such as inferiority and loneliness among young people (Kochel et al., 2012). What's worse, these suffered campus bullying youth will feel dissatisfied with school life, and their sense of belonging and identification with the school is gradually reduced, which will eventually weaken their well-being. It can be seen that the changes in students' subjective well-being from campus safety environment may be influenced by their school belonging.

### Definition of School Bullying

In general, school bullying is considered a disgusting specific attack (Olweus, 1993). With this kind of behavior, the bully usually intentionally and continuously hurt others, and make the victim feel some discomfort (Olweus, 1993). Conceptually, bullying on campus mainly resorts to systematic abuse of violence, which makes the power relationship between the bully and the victim in an unequal state (Woods and Wolke, 2004). In form, regular bullying can be physical (such as beating, punching, kicking, etc.), or verbal (such as giving others a nickname, mocking, etc.) and interpersonal (such as spreading rumors, social exclusion, or other forms of public humiliation, etc.). It is worth noting that, recent years with the widespread use of information and communication technologies, cyberbullying implemented through digital devices and tools has also become a new form of school bullying (Smith et al., 2008; Hinduja and Patchin, 2010). Moreover, in specific areas of evaluation on school bullying, since the launch of Programme for International Student Assessment (PISA) of 2015 test, the assessment on school bullying started a practical attempt, which mainly asks students about their experiences of bullying-related behaviors in school, and measures their performance on three types of bullying, namely physical bullying, verbal bullying, and interpersonal bullying (OECD, 2017).

### School Bullying and Subjective Well-being

To a certain extent, incorporating the experiences of students being bullied into the research scope of well-being will help to systematically explore the main sources and structures of adolescents' subjective well-being, thus crossing existing research boundaries (Goswami, 2014). The concept of subjective well-being covers all perceptions of each person's own lives and areas of life, that is, it is a combination of all kinds of positive feelings and negative feelings (Diener et al., 1999). Normally, students' subjective well-being are mainly stem from positive emotional experience (Diener, 2000). On the contrary, students' perception of school violence (such as bullying) is always closely related to the decline in their school life satisfaction and subjective well-being (Gavidia et al., 2014).

In fact, the current research on the impact of campus bullying on well-being is inconclusive. Some qualitative studies have pointed out, when individuals grow up, if they find themselves surrounded by bullying behaviors, such as physical harm, social exclusion, and verbal violence, individuals are more likely to have more depression, anxiety, nervousness, stress, fear, and other emotions, which brings them a more negative sense of happiness (Altun and Baker, 2010). At the same time, many empirical studies demonstrate that school bullying behavior has a great negative impact on students' subjective well-being. As Tiliouine's latest research shows that, in Algeria, 8, 10, and 12-year-old students have been subjected to various degrees of physical assault and social exclusion and other violent injuries, and the incidence of school bullying have a strong negative impact on students' overall life satisfaction (Tiliouine and Habib, 2015). And some other international survey results have also found that the experience of students being bullied at school will significantly reduce their subjective well-being (Olweus and Breivik, 2014). Nevertheless, some studies have reached inconsistent conclusions, they found that students' life satisfaction is not affected by cyberbullying (Moore et al., 2012). This may be due to the lack of suitable theory to help explain and test the possible complex relationship between these two (Bacete et al., 2014). Therefore, the academia should conduct more research to gain a deeper understanding of the connection between school bullying and subjective well-being. The comparison found that there is also a lack of systematic empirical research in mainland China, which makes it difficult to provide targeted information for educational decision makers to formulate interventions and governance programs for school bullying.

### School Bullying, School Belonging, and Subjective Well-Being

From the existing literature, amid the many factors that affect that affect adolescents' subjective well-being, students' sense of school belonging and school bullying experience are two aspects of mutual inhibition (Xu J., 2017). On the one hand, violent behaviors such as school bullying can have a negative impact on students' psychological safety perception and which even lead to school terrors, and these bullying victims no longer believe that the school is a safe place, and they often feel fear, depression, loneliness, and loss, which will weaken their sense of belonging at school. On the other hand, the positive school atmosphere and interpersonal relationships will greatly reduce frequency of school violence such as bullying (Brookmeyer et al., 2006). Therefore, most anti-bullying interventions are based on the school environment or school atmosphere, and students' positive perception of the school (such as school belonging) can be considered as an important factor that interferes with the occurrence of school bullying (Laufer and Harel, 2003). Not only that, among the “near-end sources” of adolescents' subjective well-being, the sense of students' belonging who are greatly affected by the school environment is also the main component of their psychological dimensions (OECD, 2017). In the process of pursuing subjective well-being, students tend to seek stronger social connections and collective value identification. As early as the 1940s, the sense of belonging had appeared in Maslow's hierarchy of needs theory (Maslow, 1943). Based on this theory, the need to belong and love is a need that everyone wants to be accepted, loved, cared for, encouraged, and supported by others or groups. The sense of belonging a need that arises after the physiological needs and security needs are relatively satisfied, including being loved by others and love others, and thus the sense of school belonging mainly refers to the recognition of peers and the love for classes and schools. In a sense, school belonging is a long-term school interpersonal relationship based on trust, recognition, and support (Baumeister and Leary, 1995). In the field of international evaluation, school belonging refers to the combination of students' positive perceptions and negative feelings in terms of being recognized by others and interpersonal communication (OECD, 2019). On the whole, although the research results on the correlation between bullying on campus and subjective well-being are not uniform, most researchers still believe that school bullying may lead to students' subjective well-being decline through damage to the sense of school belonging (Goldweber et al., 2013). As a result, the current clarification of how school belonging can mitigate the possible negative impact of school bullying on subjective well-being will help educators develop more effective bullying intervention plans in the future, and is also of great benefit in enhancing students' subjective well-being.

## Analysis Framework

For a long time, schools have been the main places where young people experience the potential impact of education. However, in the existing school education environment, a social and cultural phenomenon often occurs, that is, campus bullying, which actually constitutes a negative part of the school's social conditions (Gini, 2006). Usually, campus violence such as bullying does have a negative impact on students' sense of school belonging and subjective well-being to some extent. It not only hinders the interpersonal communication between students and their peers, as well as their belonging and identification with their school (Osterman, 2000), but also directly weaken the satisfaction of students' school life and subjective well-being (Altun and Baker, 2010). In addition, some empirical studies have also found that a harmonious campus culture (such as students' subjective well-being is generally high, and full understanding and recognition of school culture) can reduce the probability of aggression and violence, including school bullying (Goldstein et al., 2008).

At the same time, many studies have shown that there is a positive correlation between students' sense of school belonging and their subjective well-being. Students with a high sense of school belonging will always experience more positive emotions, so they are more satisfied with school life, and they also feel happier (Du, 2009; Wang, 2018). In addition, some existing studies have also found that the effect of campus bullying on subjective well-being is not only reflected in the direct impact, but may also play a role indirectly through some other school climate variables. For example, some evidence revealed that in the evaluation of the subjective well-being of young people in school, it is mainly through the simultaneous perception of the positive components (belonging, identity, etc.) and negative components (bullying, discrimination, etc.) which come from their school life experience and their interpersonal communication, and this comprehensive perception process will eventually form their subjective well-being (Huebner et al., 2000). On the one hand, when victims experience campus bullying, they will directly produce more negative emotions, which leads to a decline in their satisfaction with school life. On the other hand, when school bullying occurs, it will first weaken the victim's understanding and identification with the school, and then reduce their sense of school belonging, and their subjective well-being will also decline at the same time (Tian, 2007). Therefore, on the whole, students' school belonging can establish an intermediary mechanism between school bullying and subjective well-being. Meanwhile, the current research on the mediating role of school belonging will also help prevent, intervene, and solve the current problem of campus bullying.

Worldwide, teachers, parents, schools, and education policy makers have been actively looking for solutions to bullying on campus (Phillips, 2007), but few studies have systematically tested the association mechanism between school bullying and subjective well-being. Moreover, China's educational department has also implemented a series of long-term mechanisms to improve and prevent bullying in primary and middle school students, thereby effectively responding to school violence such as bullying and helping students improve mental health, as well as school belonging and well-being (MOE, 2017). In view of this above, this study focuses on exploring the relationship between school bullying and students' subjective well-being in a school environment in mainland China, and further examines the mediating role of school belonging. Specifically, the research will focus on the following issues:

(1) What is the correlation between school bullying and students' subjective well-being?
(2) Can school belonging take an intermediary role in the relationship between bullying on campus and subjective well-being?

This research mainly uses samples of middle school students over 15 years old who participated in PISA2018 from mainland China (Beijing, Shanghai, Jiangsu, and Zhejiang) to analyze and solve the above issues. Although the these four places represent the relatively advanced education and economic level of mainland China, they can provide a reference for the study of school bullying in other similar provinces in the mainland to a certain extent.

## Objects and Methods

### Participants

This research uses PISA2018 data provided by the Organization for Economic Cooperation and Development (OECD). Programme for International Student Assessment is an international student assessment project that is implemented once every 3 years. It aims to evaluate the effectiveness of the school education system by testing students in three main disciplines, namely science, mathematics, and reading (OECD, 2016). Specifically, the PISA test in China adopts a two-stage sampling method. First, in the first stage, the OECD will entrust international contractors to select sample schools using the Probability Proportional to Size (PPS) method, these sample schools will cover urban and rural schools, ordinary middle schools, vocational middle schools, middle and high schools, and schools of different running level, public schools and private schools. Then in the second stage, the PISA National Center will use the software provided by OECD to randomly select a certain number of students from the sample schools, within the same school, every student who meets the PISA conditions has the same probability of being selected, and will not be affected by student performance, etc. Finally, the project team selected the sample of middle school students over 15 years old who participated in PISA2018 from mainland China (B-S-J-Z-China), namely a total of 12,058 Chinese student participants represented by Beijing, Shanghai, Jiangsu, and Zhejiang, which is the main source of data for this study. In addition, in order to obtain relatively objective and effective analysis data, the researcher eliminated the divorced values (such as “Valid Skip,” “Not Applicable,” “Invalid,” and “No Response”) in students' response performance on campus bullying, school belonging, and subjective well-being drive from the Chinese student sample database. And after excluding invalid participants, there are 11,631 valid samples left. Among them, there are 6,045 boys (52%) and 5,586 girls (48%); 26 students in grade 7 (0.2%), 177 students in grade 8 (1.5%), 3,942 students in grade 9 (33.9%), 7,352 students in grade 10 (63.2%), and 127 students in grade 11 (1.1%), 7 students in grade 12 (0.1%); 2,231 (19.2%) 15-year-old students, and 9,400 (80.8%) 16-year-old students^1^.

### Instruments

The research instruments of this study both are mainly derived from the PISA2018 test, including the school bullying questionnaire, the sense of school belonging questionnaire, and subjective well-being indicators.

#### School Bullying Questionnaire

Since PISA 2015 has launched, the school bullying assessment has inspected students about their experiences of bullying-related behaviors in schools, and mainly investigated the frequency of three regular types of bullying, respectively, are physical bullying, interpersonal bullying, and verbal bullying. Because campus bullying is often repetitive, periodic and continuous, so the PISA 2018 test also focuses on the frequency of the above three types of bullying experienced by students in the 12 months before the test. From the whole questionnaire, there are a total of six items with similar topics, which are used to measure the basic situation of bullying on campus, such as “Other students deliberately excluded me,” “Other students teased me,” “I was threatened by other classmates,” “Other classmates took away or destroyed things that belonged to me,” “I was beaten or pushed by other students,” “Other students spread rumors about me,” and so on, these six items are all of Likert's four-level dimension scale. The item options range from “never or almost never” to “once or more per week,” each of which is counted as 1–4 scores, respectively. By computing the average score of the six items, the higher the score obtained, the higher the frequency of school bullying. In addition, in this study, the researcher will use the average score of the students to answer these items as the main measure of campus bullying variables, thereby synthesizing a comprehensive index of school bullying. The survey results show that the reliability of the scale is good, and the internal consistency coefficient is 0.840.

#### The Sense of School Belonging Questionnaire

The results of existing international assessments have shown that the stronger the sense of students' school belonging in middle school, the more positive their learning attitudes can be enhanced. Therefore, the PISA 2003 Project Team began to pay attention to the systematic investigation of school belonging of middle school students, and correspondingly designed the “*Secondary School Belonging Scale for Middle School Students*.” The questionnaire is also consists of six items, which are mainly divided into two dimensions, namely positive feelings and negative feelings, and the whole is used to measure the students' school belonging. Among them, the positive feelings are composed of items such as “I can easily make friends at school,” “I feel I belong to the school,” “Other classmates seem to like me,” and other topics, while the negative feelings are consist of “I look like an outsider in school,” “I feel embarrassed and disappointed in school,” “I feel lonely and lost at school,” and other similar topics, these six items are all of Likert's four-level dimension scale. The item options range from “strongly agree' to “strongly disagree,” each of which is counted as 1–4 scores, respectively.

In order to enhance the education system to monitor changes in the quality of interaction between students and schools, the PISA 2018 Project Team continues to use the questionnaire items from previous years, and all item options ranging from “strongly agree” to “strongly disagree,” with a value of 1–4 points, respectively. In terms of negative feelings, the degree of isolation, loss, and loneliness that students feel at school will decrease as their score increases. For example, in the response of “I look like an outsider in school,” If the student chooses “strongly agree,” it is assigned a value of “1,” and if they choose “strongly disagree,” it is assigned a value of “4.” The higher the student's score, the weaker the sense of isolation and the stronger the sense of school belonging. On the contrary, for positive feelings, the existing scoring method indicates that the higher the student's score, the weaker the attitude toward friends, the sense of self-existence and self-identity in school, and the lower the sense of school belonging. In order to facilitate the overall comparison of the students' sense of school belonging, this study performed reverse scoring processing on the item options of the positive feeling dimension. If the student chooses “strongly disagree,” it is assigned a value of “1,” and if they choose “strongly agree,” it is assigned a value of “4.” The higher the student's score, the stronger his attitude toward friendship and sense of school belonging. To sum up, under the adjustment of the scoring method, the higher the scores of students on all questions answered, the stronger their sense of school belonging. The survey results show that the reliability of the scale is also good, and the internal consistency coefficient is 0.832.

#### Subjective Well-Being

In the PISA 2018 test, the evaluation of youth well-being mainly covers “four dimensions” and “two indicators.” The “four dimensions” are life well-being, self-well-being, on-campus well-being and off-school well-being, and the “two indicators” refer to objective indicators and subjective indicators. Meanwhile, the subjective indicators can also be divided into three aspects of perception, emotion, and satisfaction (Li G. et al., 2017). In this study, the researcher specifically used Subjective Well-being Index (SWBP^2^) as the main measure of students' subjective well-being, which is formed by combining all the subjective indicators of the above four dimensions.

### Data Analysis Model

In order to analyze the indirect effect of school bullying on students' subjective well-being through school belonging, this study constructed a mediation effect model (formula is as follows):


(1) $$\begin{array}{r} \text{Y}=\text{cX}+{\text{e}}_{1} \end{array}$$

(2) $$\begin{array}{r} \text{M}=\text{aX}+{\text{e}}_{2} \end{array}$$

(3) $$\begin{array}{r} \text{Y}={\text{c}}^{\prime }\text{X}+\text{bM}+{\text{e}}_{3} \end{array}$$

**Figure 1:**
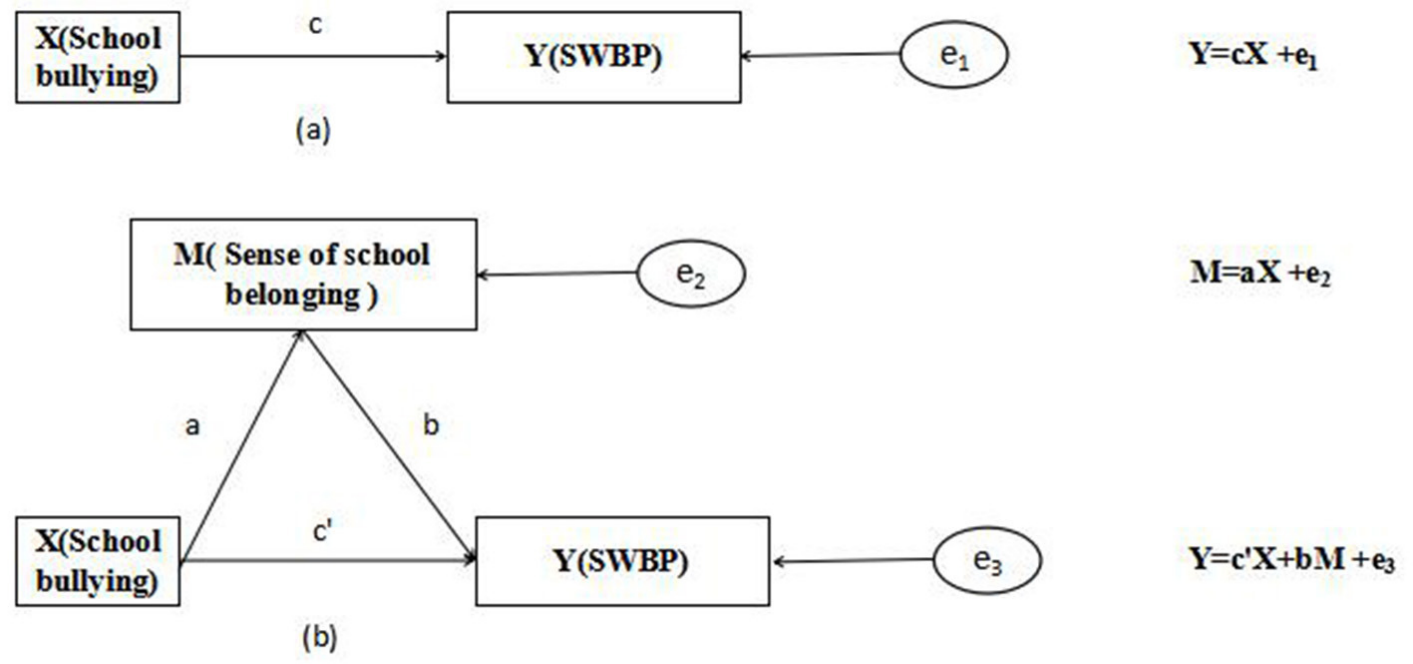
The setting of the mediating effect model.

Among the formula, Y is the student's subjective well-being, X is campus bullying, and M is school belonging. The coefficient c of formula (1) is the total effect of school bullying on students' subjective well-being; The coefficient a of formula (2) is the effect of school bullying on the sense of school belonging; The coefficient b of formula (3) is the effect of school belonging on students' subjective well-being after controlling the impact of school bullying; The coefficient c' is the direct effect of school bullying on students' subjective well-being after controlling the influence of school belonging (See Figure 1 for details). At this time, the mediation effect of school belonging is equal to ab, and the relationship between intermediary effect, direct effect and total effect is as follows:


$$\begin{array}{l} \text{Total effect}\left(\text{c}\right)=\text{direct effect}\left({\text{c}}^{\prime }\right)+\text{indirect effect }\left(\text{ab}\right) \end{array}$$

## Results and Analysis

### Descriptive Statistics and Correlation Analysis

#### Differences in Gender and Age of Related Variables

Through independent sample *t*-test^3^, it was found that there are certain differences in school bullying experience and subjective well-being of students of different genders (*t* = −19.268, *p* < 0.01, *d* = 0.36; *t* = 10.528, *p* < 0.01, *d* = 0.19), but there is no gender difference in the sense of school belonging. Comparatively speaking, boys experience bullying on campus more frequently than girls, and their subjective well-being is also lower than girls. From the perspective of age, there is no age difference between students' bullying experience and subjective well-being, but there is a certain difference in their sense of school belonging (*t* = 5.545, *p* < 0.01, *d* = 0.13). Among them, 15-year-old students have a higher sense of school belonging than 16-year-old students (see Table 1).

**Table 1:** Differences in gender and age of related variables.

**Variables**	**Gender**		**Age**	
	**Male**	**Female**	**15 years old**	**16 years old**
School bullying	1.34	1.18	1.27	1.26
	(0.528)	(0.349)	(0.457)	(0.458)
School belonging	2.96	2.94	3.01	2.94
	(0.534)	(0.564)	(0.573)	(0.574)
SWBP	0.02	0.19	0.11	0.10
	(0.925)	(0.842)	(0.906)	(0.885)

*The value in brackets is the standard deviation*.

#### Correlation Coefficient Between Related Variables

The results show that school bullying is significantly negatively correlated with the sense of school belonging and subjective well-being. The more frequent school bullying students experience, the lower their sense of school belonging and subjective well-being. In addition, gender is significantly positively correlated with school bullying, but negatively correlated with subjective well-being, while age is significantly negatively correlated with the sense of school belonging (see Table 2).

**Table 2:** Correlation coefficient between related variables (*n* = 11,631).

**Variables**	**1**	**2**	**3**	**4**	**5**
1 Gender	1	0.004	0.173^**^	0.018	−0.097^**^
2 Age	0.004	1	−0.007	−0.053^**^	−0.003
3 School bullying	0.173^**^	−0.007	1	−0.334^**^	−0.192^**^
4 School belonging	0.018	−0.053^**^	−0.334^**^	1	0.419^**^
5 SWBP	−0.097^**^	−0.003	−0.192^**^	0.419^**^	1

* *p < 0.05*,

** *p < 0.01*,

*** *p < 0.001*.

### The Mediating Effect Test

#### The Size of Mediating Effect

**Figure 2:**
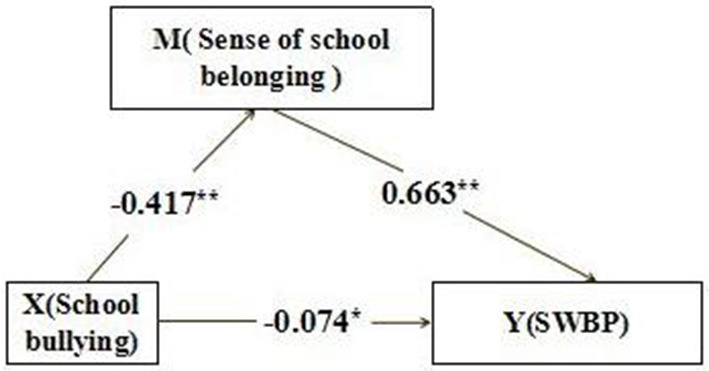
The mediating effect of school belonging between school bullying and subjecting well-being.

Considering the nesting problem of the data in this study, the researcher calculated the intra-class correlation coefficient between the student level and the school level through the null model test of the direct effect of the independent variable (school bullying) on the dependent variable (subjective well-being), and the ICC was calculated to be 0.01. Judging from the existing literature on mediation effects, it is generally believed that multilevel analysis is not considered if ICC is lower than 0.06 (Wen, 2009), so the researchers chose Bias-corrected non-parametric percentile Bootstrap method with relatively high statistical power to perform simple mediation test. Compared with other mediating effect testing methods, the main advantage of the Bootstrapping method is that it does not require distribution assumptions, so it avoids the problem in product of coefficients testing which violates distribution assumptions (Williams and Mackinnon, 2008). In this study, the specific operation is checked by the SPSS macro program PROCESS (Hayes, 2013), and the model 4 of PROCESS is selected to test the mediating effect of school belonging. Detailed results are shown in Table 3 and Figure 2.

**Table 3:** The direct effect of school bullying and the mediating effect of school belonging^a^.

**Path**	**Effect**	**Boot SE**	**Boot LLCI**	**Boot ULCI**
School bullying → School belonging → SWBP	−0.276	0.011	−0.298	−0.256
Direct effect	−0.074	0.018	−0.108	−0.039

a *Boot SE, Boot LLCI, Boot ULCI are the standard error estimated by Bootstrap method, the lower limit, and upper limit of 95% confidence interval*.

#### The Mediation Effect Model Test Results

After controlling the gender and age of middle school students, the mediating effect of school belonging is significant, accounting for 78.9% of the total effect, which indicate that regardless of gender and age, school bullying can affect student's subjective well-being through school belonging. In addition, after controlling the impact of school belonging, the direct impact of school bullying on students' subjective well-being is still significant, accounting for 21.1% of the total effect. All in all, the negative impact of school bullying on students' subjective well-being is through play a role by interfering with the students' school belonging; On the other hand, bullying behavior on campus can also directly restrict the acquisition of subjective well-being of middle school students (see Table 4).

**Table 4:** The mediation effect model test results.

**Regression equation**		**Overall fitting index**			**Significance of regression coefficient**			
**Outcome variables**	**Predictor variables**	***R***	***R^**2**^***	***F***	**β**	**Boot LLCI**	**Boot ULCI**	***t***
School belonging	Gender	0.347	0.120	529.518^***^	0.086	0.067	0.106	8.899^***^
	Age				−0.078	−0.102	−0.054	−6.419^***^
	School bullying				−0.417	−0.438	−0.396	−39.329^***^
SWBP	Gender	0.434	0.189	674.798^***^	−0.175	−0.204	−0.145	−11.518^***^
	Age				0.043	0.006	0.080	2.276^*^
	School bullying				−0.074	−0.108	−0.039	−4.205^***^
	School belonging				0.663	−0.204	−0.145	45.964^***^

* *p < 0.05*,

** *p < 0.01*,

*** *p < 0.001*.

## Discussions

### Different Manifestations of School Bullying, School Belonging, and Students' Subjective Well-Being in Different Genders and Ages

Many studies have pointed out that there is a gender difference in the frequency of school bullying as well as subjective well-being. Usually boys have higher rates of experience campus bullying than girls (Huang, 2017; Hu and Li, 2018), and their subjective well-being is also relatively lower (Ding, 2003; Xu, 2017). To some extent, this may be influenced by the expectations of men and women's social roles, namely the girl tend to be more abide by social norms, while boys are more prone to rebellion and appear interpersonal conflicts (Vargün, 2016), so boys are more likely to experience bullying in terms of physical and verbal, and thus their subjective well-being in school will be lower than that of girls. The results of this study also demonstrate that there are gender differences in the frequency of school bullying and subjective well-being of middle school students in mainland China. Chinese boys experience school bullying more frequently than girls, and boys have lower subjective well-being than girls. In addition, this study also found that school belonging of middle school students is gradually decreasing with age, which is consistent with the research conclusions of some scholars (Li and Yang, 2006; Xu and Zheng, 2008). It is that with the increase of enrollment age, middle school students' learning pressure and peer competition will also be strengthened, which affects their identification with the school and the maintenance of peer relationships, which in turn will weaken the students' sense of school belonging. In summary, for middle schools in mainland China, it is particularly necessary to actively pay attention to the gender differences in the subjective well-being of students caused by school bullying and the weakening of school belonging. Meanwhile, parents and teachers should also particularly focus on guiding boys to abide by school discipline and school rules, so as to promote the self-identity and interpersonal attitudes of senior middle school students as much as possible.

### The Mediating Effect of School Belonging

This study also found that school bullying behavior can significantly inhibit the subjective well-being of victims in middle school. The higher the frequency of bullying behaviors encountered by students, the lower their personal subjective well-being will be, which again highlights the importance of the campus safety environment for the acquisition of subjective well-being of middle school students. At the same time, this study further explores the intermediary mechanism between school bullying and subjective well-being. The results reveal that school belonging is a mediator of the connection between school bullying and subjective well-being. The school bullying not only directly restricts the acquisition of middle school students' subjective well-being, but also indirectly weakens their subjective well-being by interfering with their sense of school belonging.

The school environment has always been the main place for middle school students to learn and live, so its impact on shaping the mental health of young people in schools has also become more prominent. In the school environment in which middle school students are located, students' sense of school belonging and bullying experience on campus are the main near-end factors that affect their subjective well-being, and they inhibit each other. This study also shows that students who experience more school bullying tend to have less sense of identity and belonging at school, and they will gradually lose trust in their school. In addition, the results of this research further confirm the existing conclusion that the improvement of school belonging can not only help teenagers develop good healthy behaviors, but also greatly enhance their subjective well-being (Du, 2009). It can be seen that the influence of school bullying and school belonging on students' subjective well-being may be superimposed, and thus the school bullying behavior can greatly weaken students' sense of school belonging, and ultimately indirectly affect their subjective well-being.

## Implications and Limitations

### Implication and Significance

From the results of this study, the negative impact of school bullying on the subjective well-being of victims is mainly indirectly played by weakening their sense of school belonging, and this mediating effect is as high as 78.9%. It can be seen that the addition of the school belonging helps the academic community to systematically clarify the mechanism through which the phenomenon of campus bullying affects the subjective well-being of middle school students. At the same time, compared with the negative effects of bullying on campus, school belonging is indeed a near-end positive factor that affects students' subjective well-being in the school environment, and this important discovery provides a new direction for the formulation of school bullying prevention policies in primary and secondary schools around the world, that is, we need to focus on students' school belonging, and comprehensively enhance their understanding and recognition of the school's culture. More importantly, for Chinese primary and secondary schools which fully emphasize collective cultural identity, it is more necessary to cultivate a sense of school belonging in a targeted manner. In particular, it is also necessary to pay attention to the stage changes of students' school belonging in different ages, so as to fully respect their individuality and personality characteristics. On the other hand, the prevention and treatment of school bullying in China can also start from the class collective, the education system should actively carry out various forms of school themed cultural activities, build good peer relationships and form class cohesion, and create a relaxed and harmonious learning atmosphere and competitive environment. All in all, the continuous strengthening of school belonging in campus environment can alleviate the negative impact of school bullying on the subjective well-being of victims to a certain extent.

### Limitations and Deficiencies

Meanwhile, this research still has some deficiencies. First of all, although the PISA sampling adopts a relatively scientific PPS sampling method, and schools with different backgrounds and levels (such as school location, school type, school section, school running level, and school running property) are included in the test scope as far as possible, but the main source of the participants is still a sample of students from areas in mainland China where education and economy are relatively developed, so some conclusions are difficult to generalize to middle school students in all provinces. For example, there may also be gender differences in students' school belonging in different provinces; Secondly, in the selection of the test methods of the mediation effect, due to the relatively small intra-class correlation coefficient between the student level and the school level, this study mainly uses the Bootstrap method to perform the simple mediation effect test procedure. However, it cannot be ignored that the environment and influence process of campus bullying in middle schools are relatively complicated, and the mediation model at the student level may not reveal the overall information. On the other hand, the reference value of ICC cannot be used as the only criterion for whether choosing a multilevel mediating effect model. Moreover, compared to student's academic achievement, the inter-school differences of psychological factors such as school belonging and subjective well-being are not large, so the future research still needs to consider the multilevel mediating effect between school bullying and subjective well-being to deepen the impact of school bullying influencing mechanism of subjective well-being; Finally, this study is still a cross-sectional study, so it has not been able to thoroughly explore the environment and influencing conditions of the victims experiencing bullying on campus in different periods and stages. For instance, this research is impossible to investigate whether school belonging still plays an intermediary role between school bullying and the subjective well-being before and after the Chinese Ministry of Education promulgated the important act of *the plan of strengthen comprehensive management of bullying in primary and secondary school* (MOE, 2017), as well as the changes in the intermediary role of students' school belonging of different grades. Therefore, follow-up tracking design can be used for the developmental research.

## Conclusions

This study used the valid test data of 11,631 middle school students who participate in PISA 2018 from mainland China, which constructed a mediating effect model, and systematically explore the mechanism of influence between school bullying behavior and middle school students' subjective well-being, and also clarify some of the intermediary role played by the sense of school belonging between school bullying and students' subjective well-being, the main findings are as follows:

First, there are gender differences in the frequency of middle school students who encounter campus bullying as well as their subjective well-being, but there is no difference in their school belonging between boys and girls. Among them, boys experience school bullying more frequently than girls, and boys' subjective well-being is also lower than girls. There is also no age difference between the school bullying experience and subjective well-being of middle school students, but there is a certain difference in their school belonging. School belonging of 15-year-old students is higher than that of 16-year-old students.

Second, school bullying has a significantly negative predictive effect on school belonging and subjective well-being of middle school students. The more frequent school bullying behaviors the victims experience, the lower their school belonging and subjective well-being.

Finally, students' sense of school belonging plays a part of the intermediary role in the connection between the victims' school bullying experience and their subjective well-being, and the mediating effect accounts for 78.9% of the total effect.

## Data Availability Statement

Publicly available datasets were analyzed in this study. This data can be found here: https://www.oecd.org/pisa/data.

## Author's Note

The 67th General Funding Project of China Postdoctoral Science Foundation “Model Construction and Empirical Research on Evaluation of Mathematical Core Literacy of Middle School Students” (Project Number: 2020M670006).

## Author Contributions

All authors listed have made a substantial, direct and intellectual contribution to the work, and approved it for publication.

## Conflict of Interest

The authors declare that the research was conducted in the absence of any commercial or financial relationships that could be construed as a potential conflict of interest.

## Publisher's Note

All claims expressed in this article are solely those of the authors and do not necessarily represent those of their affiliated organizations, or those of the publisher, the editors and the reviewers. Any product that may be evaluated in this article, or claim that may be made by its manufacturer, is not guaranteed or endorsed by the publisher.

## Footnotes

^1^In this study, 15 years and 16 years mean the average age.

^2^In order to express the subjective well-being index conveniently, SWBP is used in this article for abbreviation, the same below.

^3^*d* represent the effect size of the t-test.
